# Supplementary material for: A Strategy for Identification and Structural Characterization of Compounds from Plantago asiatica L. by Liquid Chromatography-Mass Spectrometry Combined with Ion Mobility Spectrometry
Source: Molecules. 2022 Jul 4;27(13):4302. doi: 10.3390/molecules27134302 (PMC9268332; doi:10.3390/molecules27134302)
Supplement: Supplementary file 1 [file molecules-27-04302-s001.zip › molecules-1745264-supplementary.pdf]

molecules

MDPI

Supplementary Materials

Article

A Strategy for Identification and Structural Characterization of Compounds from *Plantago asiatica* L. by Liquid Chromatography-Mass Spectrometry Combined with Ion Mobility Spectrometry

Hongxue Gao [1,2], Zhiqiang Liu [1,2,3,*], Fengrui Song [1], Junpeng Xing [1], Zhong Zheng [1] and Shu Liu [1,*]

1 Jilin Provincial Key Laboratory of Chinese Medicine Chemistry, Changchun Institute of Applied Chemistry, Chinese Academy of Sciences, Changchun 130022, China; mslab34@ciac.ac.cn (H.G.); songfr@ciac.ac.cn (F.S.); xjp@ciac.ac.cn (J.X.); zhengzh@ciac.ac.cn (Z.Z.)
2 Institute of Applied Chemistry and Engineering, University of Science and Technology of China, Hefei 230029, China
3 State Key Laboratory of Electroanalytical Chemistry, Changchun Institute of Applied Chemistry, Chinese Academy of Sciences, Changchun 130022, China
* Correspondence: liuzq@ciac.ac.cn (Z.L.); mslab20@ciac.ac.cn (S.L.); Tel.: +86-431-85262613 (S.L.); Fax: +86-431-85262044 (Z.L.)

Abstract: *Plantago asiatica* L. (PAL) as a medicinal and edible plant is rich in chemical compounds, which makes the systematic and comprehensive characterization of its components challenging. In this study, an integrated strategy based on three-dimensional separation including AB-8 macroporous resin column chromatography, ultra-high performance liquid chromatography–quadrupole time-of-flight mass spectrometry (UHPLC-Q-TOF MS), and ultra-high performance liquid chromatography-mass spectrometry with ion-mobility spectrometry (UHPLC-IM-MS) was established and used to separate and identify the structures of compounds from PAL. The extracts of PAL were firstly separated into three parts by AB-8 macroporous resin and further separated and identified by UHPLC-Q-TOF MS and UHPLC-IM-MS, respectively. Additionally, UHPLC-IM-MS was used to identify isomers and coeluting compounds, so that the product ions appearing at the same retention time (RT)can clearly distinguish where the parent ion belongs by their different drift times. UNIFI software was used for data processing and structure identification. A total of 86 compounds, including triterpenes, iridoids, phenylethanoid glycosides, guanidine derivatives, organic acids, and fatty acids, were identified by using MS information and fragment ion information provided by UHPLC-Q-TOF MS and UHPLC-IM-MS. In particular, a pair of isoforms of plantagoside from PAL were detected and identified by UHPLC-IM-MS combined with the theoretical calculation method for the first time. In conclusion, the AB-8 macroporous resin column chromatography can separate the main compounds of PAL and enrich the trace compounds. Combining UHPLC-IM-MS and UHPLC-Q-TOF MS can obtain not only more fragments but also their unique drift times and RT, which is more conducive to the identification of complex systems, especially isomers. This proposed strategy can provide an effective method to separate and identify chemical components, and distinguish isomers in the complex system of traditional Chinese medicine (TCM).

Keywords: *Plantago asiatica* L.; UHPLC-Q-TOF MS; ion mobility spectrometry; macroporous resin

Table S1. Compounds identified from Plantago asiatica L by MS.

No.	RT (min)	Observed m/z	Calculated m/z	Molecular formula	mDa	Major fragments m/z	Proposed compound	Class
1	0.60	195.0493	195.0505	$C_6H_{11}O_7$	-6.2	195.0396,181.0583,129.0060,98.9940, 74.9925	Gluconic acid	Organic acid
2	0.86	260.1609	260.1610	$C_{11}H_{22}N_3O_4$	-0.4	242.1490,224.1399,206.0998,192.9579,84.0431	Hydroxyplantagoamidinic acid b	Guanidine derivative
3	1.16	345.1183	345.1186	$C_{15}H_{22}O_9$	-0.8	183.0656,165.0552,139.0260	Aucubin*	Iridoid
4	1.23	240.1335	240.1339	$C_{11}H_{18}N_3O_3$	-1.7	222.1206,184.0674,84.0435	Planasine a	Guanidine derivative
5	1.41	345.1165	345.1186	$C_{15}H_{22}O_9$	-6.1	391.1240,183.0679	Aucubin*	Iridoid
6	1.42	191.0197	191.0192	$C_6H_7O_7$	2.6	191.0433,129.0414,115.0264,101.0459	Citric acid	Organic acid
7	1.46	507.1722	507.1719	$C_{21}H_{32}O_{14}$	0.6	329.1226,179.0559	6′-o-glucosylaucubin	Iridoid
8	1.55	535.1673	535.1668	$C_{22}H_{32}O_{15}$	0.9	373.3337,221.0613,193.0350,	Desacetylhookerioside*	Iridoid
9	2.03	373.1145	373.1135	$C_{16}H_{22}O_{10}$	2.7	211.0615,167.0591,149.0609,123.0354,101.0096,86.9932	Geniposidic acid	Iridoid
10	2.43	242.1353	242.1348	$C_{11}H_{20}N_3O_3$	2.1	224.1373,178.1281,84.0436	Dehydroplantagoamidinic acid B	Guanidine derivative
11	2.52	375.1291	375.1291	$C_{16}H_{24}O_{10}$	0	213.0763,169.0746,151.0634	8-epiloganic acid	Iridoid
12	2.67	535.1673	535.1668	$C_{22}H_{32}O_{15}$	0.9	373.1133,211.0610,149.0501	Desacetylhookerioside*	Iridoid
13	2.82	375.1286	375.1291	$C_{16}H_{24}O_{10}$	-1.3	161.0451,113.0241	8-epiloganic acid	Iridoid
14	2.83	461.1658	461.1659	$C_{20}H_{29}O_{12}$	-0.2	315.1081,298.0852,161.0451	Decaffeoylacteoside	Phenylethanoid glucoside
15	3.31	487.1459	487.1457	$C_{21}H_{28}O_{13}$	0.4	341.0932,179.0347,161.0240	Cistanoside F*	Phenylethanoid glucoside
16	3.57	355.1034	355.1029	$C_{16}H_{20}O_9$	1.4	193.0504,151.0395	Gentiopicroside*	Iridoid

17	4.10	224.1387	224.1399	$C_{11}H_{18}N_3O_2$	-5.4	206.0946,178.1293,84.0433	Plumbagine B	Guanidine derivative
18	4.34	179.0350	179.0344	$C_9H_7O_4$	3.4	135.0325	Caffeic acid	Organic acid
19	4.39	481.0987	481.0982	$C_{21}H_{21}O_{13}$	1.0	319.0374,166.9883,151.0294	Ampelopsin glucoside*	Flavonoid
20	4.69	355.1035	355.1029	$C_{16}H_{20}O_9$	1.7	295.0715,265.0613,235.0395,193.0500,175.0392	Gentiopicroside*	Iridoid
21	4.82	487.1467	487.1457	$C_{21}H_{28}O_{13}$	2.1	341.1013,193.0500,179.0336	Cistanoside F*	Phenylethanoid glucoside
22	4.88	685.2009	685.1985	$C_{29}H_{36}O_{16}$	3.5	623.1984,161.0455	Orobanchoside*	Phenylethanoid glucoside
23	5.09	415.1258	415.1246	$C_{18}H_{24}O_{11}$	2.9	235.0605,135.0446	Alpinoside	Iridoid
24	5.17	481.0987	481.0982	$C_{21}H_{21}O_{13}$	1.0	319.0471,166.9983,151.0400	Ampelopsin glucoside*	Flavonoid
25	5.41	655.1890	655.1880	$C_{29}H_{36}O_{17}$	1.5	492.1299,161.0232	Hellicoside	Phenylethanoid glucoside
26	5.67	665.2093	665.2087	$C_{31}H_{38}O_{16}$	0.9	503.1667	2′-acetylacteoside*	Phenylethanoid glucoside
27	5.80	465.1034	465.1033	$C_{21}H_{22}O_{12}$	0.2	303.0506,179.0349,161.0241,151.0396	Plantagoside*	Flavonoid
28	5.84	639.1930	639.1925	$C_{29}H_{36}O_{16}$	0.8	621.1794,313.1022,303.0506,161.0241,179.0349,151.0396	Suspensaside	Phenylethanoid glucoside
29	6.22	463.0864	463.0877	$C_{21}H_{20}O_{12}$	-2.8	285.0400,135.0499,164.9827	6-hydroxyluteolin-7-O-glucoside	Flavonoid
30	6.38	226.1568	226.1556	$C_{11}H_{20}N_3O_2$	5.3	208.1446,180.1443,84.0453	Plantagoguanidinic acid	Guanidine derivative
31	6.89	639.1956	639.1925	$C_{29}H_{36}O_{16}$	4.8	477.1627,315.1088,161.0252	Plantamajoside	Phenylethanoid glucoside
32	6.92	465.1050	465.1033	$C_{21}H_{22}O_{12}$	3.7	313.0939,303.0516,151.0402	Plantagoside*	Flavonoid
33	6.92	465.1050	465.1033	$C_{21}H_{22}O_{12}$	3.7	313.0939,303.0516,151.0402	Plantagoside*	Flavonoid

34	6.92	465.1050	465.1033	$C_{21}H_{22}O_{12}$	3.7	313.0939,303.0516,151.0402	Plantagoside*	Flavonoid
35	6.95	665.2081	665.2087	$C_{31}H_{38}O_{16}$	-0.9	503.1667	2′-acetylacteoside*	Phenylethanoid glucoside
36	6.98	449.1107	449.1089	$C_{21}H_{22}O_{11}$	4.0	495.1162,150.0320,134.0373	Miscanthoside*	Flavonoid
37	7.00	463.0890	463.0877	$C_{21}H_{21}O_{12}$	2.8	303.0498,257.0465,153.0180	Isoquercitrin	Flavonoid
38	7.17	303.0512	303.0505	$C_{15}H_{12}O_{7}$	2.3	181.0511	Pntahydroxyflavanone*	Flavonoid
39	7.28	593.1527	593.1506	$C_{27}H_{29}O_{15}$	3.5	447.0962,285.0394,151.0391	Kempferol rhamnoside hexoside	Flavonoid
40	7.29	447.0934	447.0927	$C_{21}H_{20}O_{11}$	1.6	285.04064,151.1350	Maritimein	Flavonoid
41	7.35	479.1202	479.1195	$C_{22}H_{24}O_{12}$	1.5	447.0934,285.0406	Nepetin-7-O-glucoside	Flavonoid
42	7.38	623.2022	623.1976	$C_{29}H_{36}O_{15}$	7.4	461.1682,315.1094,179.0357,161.0254	Acteoside	Phenylethanoid glucoside
43	7.85	639.1958	639.1925	$C_{29}H_{36}O_{16}$	5.2	477.1619,313.0938	Orobanchoside*	Phenylethanoid glucoside
44	8.05	623.2001	623.1976	$C_{29}H_{36}O_{15}$	4.0	461.1671,315.1084,179.0351,161.0251	Isoacteoside	Phenylethanoid glucoside
45	8.45	623.1992	623.1976	$C_{29}H_{36}O_{15}$	2.6	461.1663,315.1084,179.0348,161.0247	Forsythiaside	Phenylethanoid glucoside
46	8.57	303.0516	303.0510	$C_{15}H_{12}O_{7}$	2.0	177.0196,151.0400,125.0966	Pntahydroxyflavanone*	Flavonoid
47	8.64	577.1572	577.1557	$C_{27}H_{30}O_{14}$	2.6	431.0889,413.0868,269.0456,151.0057,146.9570	Rhoifolin	Flavonoid
48	9.11	477.1418	477.1402	$C_{23}H_{26}O_{11}$	3.4	311.0658	Isorhamnetin-3-O-glucoside	Phenylethanoid glucoside
49	9.16	637.2151	637.2132	$C_{30}H_{38}O_{15}$	3.0	461.1673,443.1542,315.1097,297.0935,193.0510,175.0404,	Leucoseptoside A	Phenylethanoid glucoside
50	9.35	287.0560	287.0556	$C_{15}H_{12}O_{6}$	1.4	151.0039,135.0452	Eriodictyol*	Flavonoid

51	9.35	449.1098	449.1084	$C_{21}H_{22}O_{11}$	3.1	287.0563,151.0039,135.0452	Miscanthoside*	Flavonoid
52	9.36	597.2162	597.2183	$C_{28}H_{37}O_{14}$	-3.5	161.0169	Picraquassioside C	Phenylethanoid glucoside
53	9.49	665.2065	665.2087	$C_{31}H_{38}O_{16}$	-3.3	461.1659,285.0404,161.0246	2′-acetylacteoside*	Phenylethanoid glucoside
54	9.95	421.2080	421.2074	$C_{19}H_{33}O_{10}$	1.4	289.1665,175.0405,161.0459,143.0237,113.0108,101.0244	1-octen-3-yl-primeveroside	Aliphatic alcohol glycoside
55	10.04	651.2311	651.2289	$C_{31}H_{40}O_{15}$	3.4	503.1571,337.0934,193.0511,179.0405,161.0231	Martynoside	Phenylethanoid glucoside
56	10.04	299.0567	299.0561	$C_{16}H_{12}O_6$	2.0	268.0384,161.0231,151.0073	Hispidulin	Flavonoid
57	10.38	287.0567	287.0556	$C_{15}H_{12}O_6$	3.8	151.0041,135.0453	Eriodictyol*	Flavonoid
58	10.45	285.0412	285.0399	$C_{15}H_{10}O_6$	4.6	179.0354,133.0296	Luteoline	Flavonoid
59	11.13	327.2175	327.2171	$C_{18}H_{31}O_5$	1.2	229.1449,211.1338,171.1024	Trihydroxy-octadecadienoic*	Fatty acid
60	11.53	329.2337	329.2328	$C_{18}H_{33}O_5$	2.7	211.1233,171.1028	Hydroxyoctadecanenedioc acid	Fatty acid
61	11.80	287.2229	287.2222	$C_{16}H_{31}O_4$	2.4	121.0207	Dihydroxypacmitic acid*	Fatty acid
62	12.47	255.2323	255.2324	$C_{16}H_{32}O_2$	-0.4	301.2378,197.1079	Palmitic acid	Fatty acid
63	12.73	327.2177	327.2171	$C_{18}H_{31}O_5$	1.8	197.1078,171.1026	Trihydroxy-octadecadienoic*	Fatty acid
64	14.21	313.2384	313.2379	$C_{18}H_{31}O_4$	1.6	307.1809,235.1214,211.1243,185.1076	Hydroperoxy-octadecenoic acid*	Fatty acid
65	14.21	311.2229	311.2222	$C_{18}H_{31}O_4$	2.2	201.1034,171.0915	Hydroperoxyl-octadecanedioic acid	Fatty acid
66	14.96	287.2227	287.2222	$C_{16}H_{31}O_4$	1.7		Dihydroxypacmitic acid*	Fatty acid
67	15.23	313.2349	313.2379	$C_{18}H_{31}O_4$	-9.6	277.2063,201.1030,183.1294	Hydroperoxy-octadecenoic acid*	Fatty acid

68	15.34	593.2761	593.2756	$C_{27}H_{46}O_{12}P$	0.8	315.0508,241.0132	Pi(18:3/0:0)	Glycerophos-phosp-holipid
69	15.75	313.2400	313.2379	$C_{18}H_{31}O_4$	6.7	295.2193,201.1141,171.1033	Hydroperoxy-octade-cenoic acid*	Fatty acid
70	15.86	593.2756	593.2756	$C_{27}H_{46}O_{12}P$	0	413.2106,315.0497,277.2781,241.0128	Pi(18:3/0:0)	Glycerophos-phosp-holipid
71	16.75	595.2889	595.2883	$C_{27}H_{48}O_{12}P$	1.0	279.2256,241.0045,152.9874	Pi(18:2/0:0)*	Glycerophos-phosp-holipid
72	16.85	315.2539	315.2535	$C_{18}H_{35}O_4$	1.3	297.2434,171.1026,141.1270	Dihydroxy-stearic acid*	Fatty acid
73	17.42	595.2900	595.2883	$C_{27}H_{48}O_{12}P$	2.9	279.2269,241.0062,152.9901	Pi(18:2/0:0)*	Glycerophos-phosp-holipid
74	17.49	315.2538	315.2535	$C_{18}H_{35}O_4$	1.0	297.2431,171.1026,141.1283	Dihydroxy-stearic acid*	Fatty acid
75	17.85	571.2898	571.2883	$C_{25}H_{48}O_{12}P$	2.6	255.2330,241.0123	Pi(16:0/0:0)*	Glycerophos-phosp-holipid
76	18.59	571.2909	571.2883	$C_{25}H_{48}O_{12}P$	4.6	255.2335,241.0128	Pi(16:0/0:0)*	Glycerophos-phosp-holipid
77	19.58.	597.3058	597.3040	$C_{27}H_{50}O_{12}P$	3.0	417.2408,281.2485,241.0124	Pi(18:1/0:0)	Glycerophos-phosp-holipid
78	20.09	297.2433	297.2430	$C_{18}H_{33}O_3$	1.0		Hydroxyl-octadecadi-enoic acid	Fatty acid
79	20.55	293.2121	293.2117	$C_{18}H_{29}O_3$	1.4		Hydroxy-octadeca-trienoic acid	Fatty acid
80	22.22	277.2171	277.2168	$C_{18}H_{29}O_2$	1.1		Linolenic acid	Fatty acid
81	22.24	455.3529	455.3530	$C_{30}H_{48}O_3$	-0.2	407.1740	Oleanolic acid	Triterpenoid
82	22.37	338.2700	338.2700	$C_{20}H_{37}NO_3$	0	384.2755,281.2481	N-oleoyl glycine	Fatty amide
83	22.63	455.3539	455.3530	$C_{30}H_{48}O_3$	2.0	407.1740	Ursolic acid	Triterpenoid
84	23.08	313.2381	313.2379	$C_{18}H_{33}O_4$	0.6	201.1128,171.1025	Hydroperoxy-octade-cenoicacid*	Fatty acid
85	23.20	281.2488	281.2481	$C_{18}H_{34}O_2$	2.5	96.9461	Oleic acid	Fatty acid
86	23.44	453.3369	453.3374	$C_{30}H_{46}O_3$	-1.1	407.3376	Ursonic acid	Triterpenoid

* These compounds have been detected as isomers from *Plantago asiatica L.*

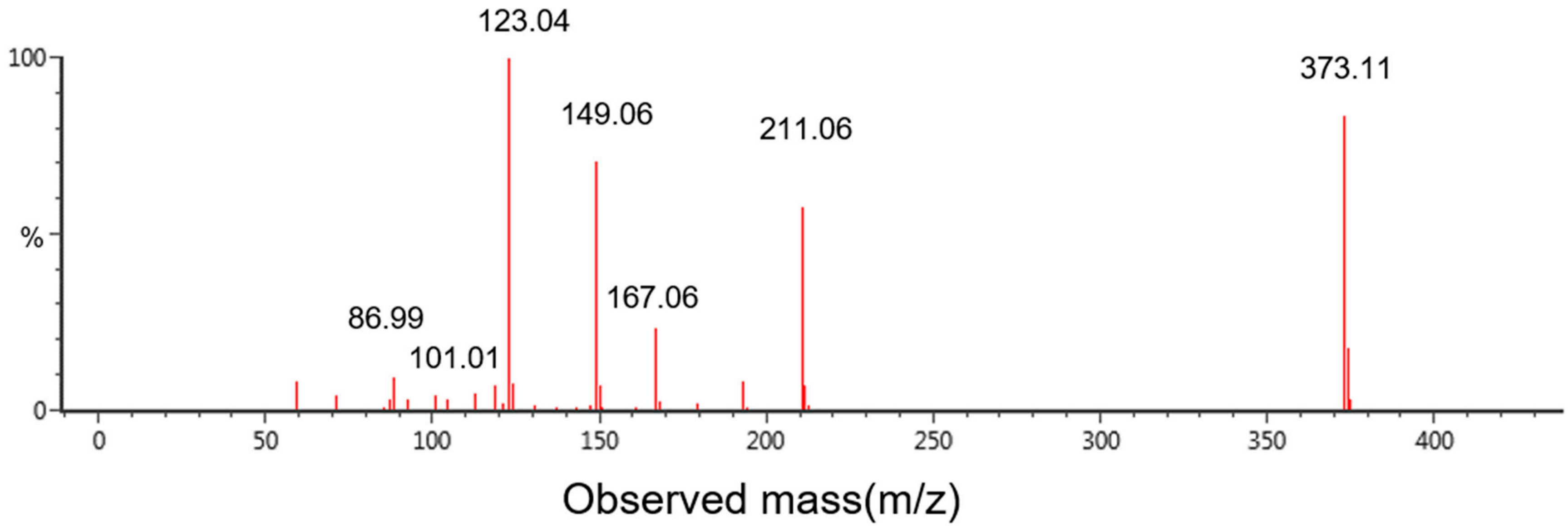

Figure S1. The fragment ion information of geniposidic acid (compound **9**) recorded by UHPLC-Q-TOF-MS.

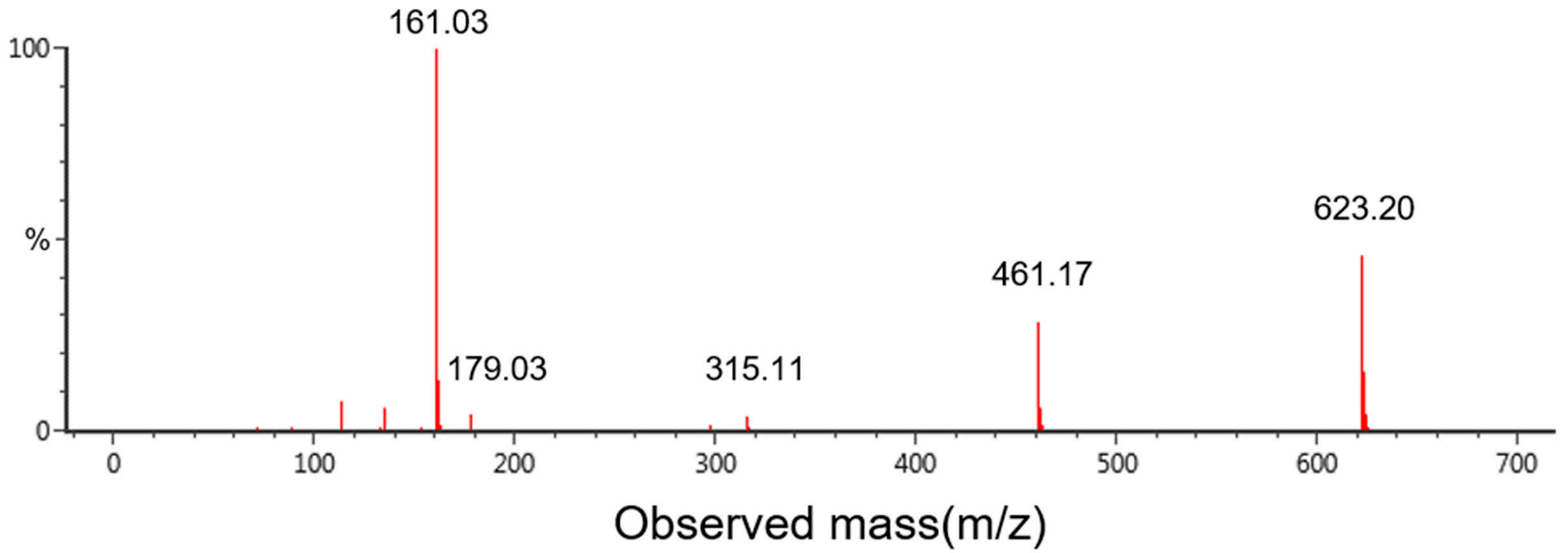

Figure S2. The fragment ion information of acteoside (compound **42**) recorded by UHPLC-Q-TOF-MS.

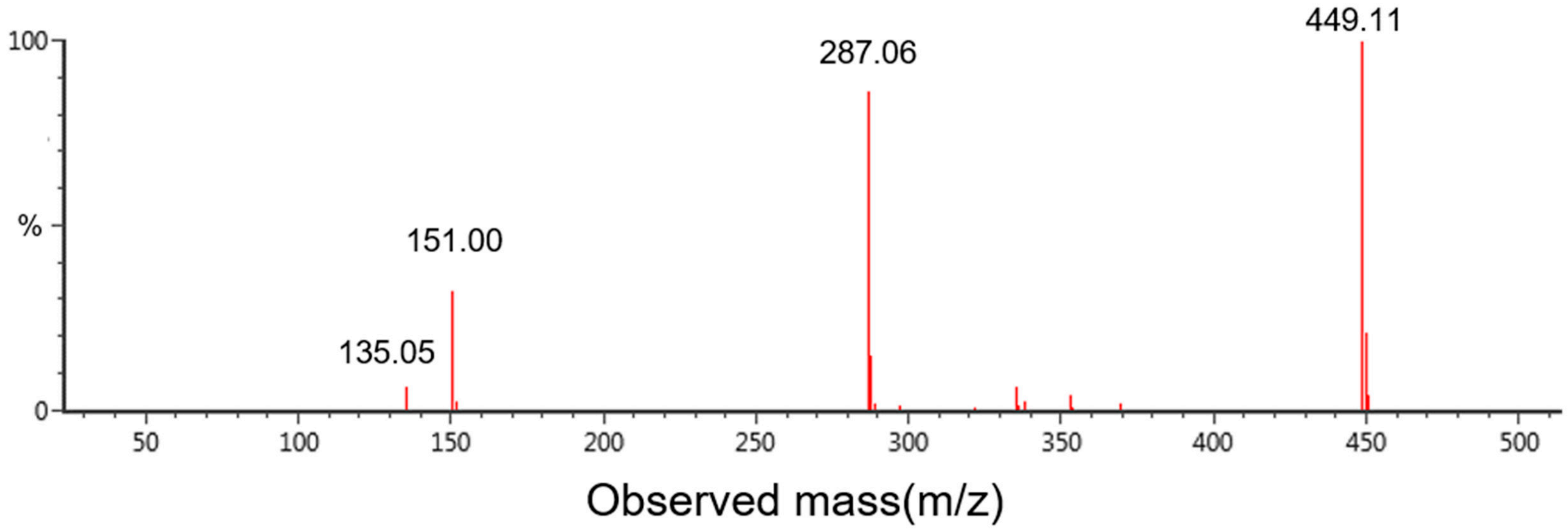

Figure S3. The fragment ion information of miscanthoside (compound **51**) recorded by UHPLC-Q-TOF-MS.
